# ERICA: prevalence of metabolic syndrome in Brazilian adolescents

**DOI:** 10.1590/S01518-8787.2016050006701

**Published:** 2016-02-02

**Authors:** Maria Cristina C Kuschnir, Katia Vergetti Bloch, Moyses Szklo, Carlos Henrique Klein, Laura Augusta Barufaldi, Gabriela de Azevedo Abreu, Beatriz Schaan, Gloria Valeria da Veiga, Thiago Luiz Nogueira da Silva, Maurício T L de Vasconcellos, e other authors

**Affiliations:** I Núcleo de Estudos da Saúde do Adolescente. Faculdade de Ciências Médicas.Universidade do Estado do Rio de Janeiro. Rio de Janeiro, RJ, Brasil; II Instituto de Estudos em Saúde Coletiva. Universidade Federal do Rio de Janeiro. Rio de Janeiro, RJ, Brasil; III Escola Nacional de Saúde Pública. Fundação Oswaldo Cruz. Rio de Janeiro, RJ, Brasil; IV Programa de Pós-Graduação em Saúde Coletiva. Instituto de Medicina Social. Universidade do Estado do Rio de Janeiro. Rio de Janeiro, RJ, Brasil; V Hospital de Clínicas de Porto Alegre. Universidade Federal do Rio Grande do Sul. Porto Alegre, RS, Brasil; VI Instituto de Nutrição Josué de Castro. Universidade Federal do Rio de Janeiro. Rio de Janeiro, RJ, Brasil; VII Escola Nacional de Ciências Estatísticas. Fundação Instituto Brasileiro de Geografia e Estatística. Rio de Janeiro, RJ, Brasil

**Keywords:** Adolescent, Metabolic Syndrome, epidemiology, Risk Factors, Cardiovascular Diseases, Cross-Sectional Studies

## Abstract

**OBJECTIVE:**

To determine the prevalence of metabolic syndrome and its components in Brazilian adolescents.

**METHODS:**

We evaluated 37,504 adolescents who were participants in the Study of Cardiovascular Risks in Adolescents (ERICA), a cross-sectional, school-based, national study. The adolescents, aged from 12 to 17 years, lived in cities with populations greater than 100,000 inhabitants. The sample was stratified and clustered into schools and classes. The criteria set out by the International Diabetes Federation were used to define metabolic syndrome. Prevalences of metabolic syndrome were estimated according to sex, age group, school type and nutritional status.

**RESULTS:**

Of the 37,504 adolescents who were evaluated: 50.2% were female; 54.3% were aged from 15 to 17 years, and 73.3% were from public schools. The prevalence of metabolic syndrome was 2.6% (95%CI 2.3-2.9), slightly higher in males and in those aged from 15 to 17 years in most macro-regions. The prevalence was the highest in residents from the South macro-region, in the younger female adolescents and in the older male adolescents. The prevalence was higher in public schools (2.8% [95%CI 2.4-3.2]), when compared with private schools (1.9% [95%CI 1.4-2.4]) and higher in obese adolescents when compared with nonobese ones. The most common combinations of components, referring to 3/4 of combinations, were: enlarged waist circumference (WC), low HDL-cholesterol (HDL-c) and high blood pressure; followed by enlarged WC, low HDL-c and high triglycerides; and enlarged WC, low HDL-c, high triglycerides and blood pressure. Low HDL was the second most frequent component, but the highest prevalence of metabolic syndrome (26.8%) was observed in the presence of high triglycerides.

**CONCLUSIONS:**

ERICA is the first Brazilian nation-wide study to present the prevalence of metabolic syndrome and describe the role of its components. Despite the prevalence of Metabolic Syndrome being low, the high prevalences of some components and participation of others in the syndrome composition shows the importance of early diagnosis of this changes, even if not grouped within the metabolic syndrome.

## INTRODUCTION

The increasing prevalence of overweight and obese children and adolescents, which has been observed in several countries^18^, including Brazil^15^, represent a serious public health problem in terms of the various health risks that obesity can cause, both during adolescence and adult life. Included among the already recognized cardiovascular risk factors are those that make up the so-called metabolic syndrome (MS). The following are associated with MS: enlarged waist circumference (WC), low levels of high-density lipoprotein cholesterol (HDL-c), high systemic blood pressure, high triglycerides, and high blood glucose. An individual is positively diagnosed with MS if he/she has at least three of these conditions, which are related to cardiovascular disease and can have it as one of the adverse outcomes^24^.

Various proposals are set out to define MS in children and adolescents. However, no consensus exists on what their components or cut-off points would be, which have already been defined for adults. In 2004, Ferranti et al.^10^ proposed an adaptation of the criteria created by the National Cholesterol Education Program Adult Treatment Panel (NCEP-ATPIII). According to this MS definition, the cut-off point for WC is the 70^th^ percentile^10^.

In 2007, the International Diabetes Federation (IDF) established a new concept for defining MS in children and adolescents. This concept considers increased WC measurement as the main component for defining MS. Thus, MS became being diagnosed in children aged 10 years or more when their WC was greater than or equal to the 90^th^ percentile of the curve developed by Fernandez et al.^9^, and at the same time, presenting two or more of the clinical or laboratory criteria (low HDL-c and high blood pressure, triglycerides and high glucose). For adolescents aged over 16 years, the adult IDF criteria are used^25^. In 2014, Giannini et al.^1^ ^2^ observed that using the IDF criteria resulted in lower prevalences in a sample of overweight adolescents when compared with the NCEP-ATPIII criteria. Tavares et al.^22^, during a systematic review of 15 articles performed with Brazilian adolescents, 11 of which are in the Southeast region, observed a variation of between 0% and 42.0% in MS prevalence, which depended on the diagnostic criteria used, being more pronounced in overweight individuals.

The objective of this study was to determine the prevalence of MS in Brazilian adolescents.

## METHODS

The *Estudo de Riscos Cardiovasculares em Adolescentes* (ERICA – Study of Cardiovascular Risk in Adolescents) was designed to estimate the prevalence of cardiovascular risk factors that make up MS in a representative sample of adolescents aged from 12 to 17 years. The selected adolescents were attending the seventh, eighth and ninth year of elementary school or the first, second or third year of high school, in public or private schools, studied in the morning shift and lived in Brazilian cities with populations higher than 100,000 inhabitants. These school years weres chosen to include the largest possible number of adolescents aged from 12 to 17 years, since no information base exists regarding individual adolescent units for a sample selection, but only combinations of classes and years at the schools.

The sample was stratified into 32 strata, made up of the 27 Brazilian state capital cities and five sets of the other countryside cities, with more than 100,000 inhabitants, from each of the five macro-regions of Brazil. Thus, the sample is representative for medium- and large-sized cities at the regional and national level and for the capitals and the Federal District. Clusters were selected at three levels: schools, combinations of year and shift, and classes. The sampling weights were calculated by the product of the inverse probability of inclusion at each stage of the sample selection and were calibrated considering the estimates of the adolescent population enrolled in schools located in the geographic strata based on sex and age. The analysis was adjusted to account for the sample design, which was done using statistical routines for a complex sampling that considers the sources of variability and calibration with population estimates.

The sample size calculation considered an expected MS prevalence of 4.0% in adolescents^6^, with a maximum error level of 0.9% and a 95% confidence level, as well as a 2.97 agglomeration effect. A detailed description of the sampling process can be seen in Vasconcellos et al.^23^

To estimate the prevalence of MS, biochemical analyses were performed in the plasma. The fasting time required for blood collection was 12 hours, and therefore was only done with the morning shift students who were selected in the ERICA sample. Thus, any inference from the results regarding MS can only be made for students who attend the morning shift.

The sex and age variables were obtained through a self-filled questionnaire that was completed by the respondent on an electronic data collector (PDA) which also contained other questions not used in this analysis. The age variable was confirmed using school records. The following anthropometrical variables were measured by trained researchers: WC, measurement of the circumference of the right arm (for choosing the appropriate cuff for measuring blood pressure), weight and height. These measurements were monitored throughout the collection by means of a quality control that verified their limits and distribution of digits.

Weight was evaluated using a P200M Líder^®^ scale, which has a capacity of up to 200 kg and a 50 g variation. Height was measured twice using an Altura Exata^®^ portable stadiometer with a 0.1 variation, with the mean of the two values obtained being the value taken into consideration. The weight and height measurements were used to classify the nutritional status, based on the body mass index calculation (BMI = weight/height^2^). Classifying the nutritional status involved using the BMI curves as proposed by the World Health Organization^17^ (2007) specified by age and sex. Adolescents considered to have an adequate nutritional status had a score of +1 > Z ≥ -2, overweight adolescents had a score of +2 > Z ≥ +1, and obese adolescents were those with a Z ≥ +2 score.

WC was measured using a Sanny^®^ inelastic measuring tape, which had 0.1 cm variation, at the midpoint between the bottom of the last rib and the upper curvature of the iliac crest, with the adolescent in a standing position, arms along the body and with his/her feet and abdomen relaxed.

Blood pressure was measured using a Omron^®^ 705-IT, machine, which had been validated for use in adolescents^21^. Three blood pressure measurements were taken, with the mean of the final two being used in the hypertension classification. Systemic blood pressure was considered high if the systolic blood pressure was greater than or equal to 130 mmHg, and the diastolic blood pressure was greater than or equal to 85 mmHg.

Blood sample analyses of biochemical parameters that are included in MS (HDL-c, triglycerides and glucose) were performed in only one laboratory that followed the current required quality standards for its qualification. Fasting blood glucose was analyzed with the GOD-PAP enzymatic method in the Roche modular analytical equipment. Values of 100 mg/dL or more were considered as discriminants of high blood glucose^2^. Triglycerides and HDL-c were analyzed with the enzymatic colorimetric method using Roche modular analytical equipment. Reference values published in I Guidelines of Prevention of Atherosclerosis during Childhood and Adolescence were used^20^. A full description of the ERICA methods can be found in Bloch et al.^4^

A positive MS classification was given when at least three of the below components were present, with a mandatory requirement for enlarged WC, which is in accordance with the criteria from the International Diabetes Federation^2^:

• WC

< 16 years: ≥ 90^th^ percentile

≥ 16 years, male: ≥ 90 cm

≥ 16 years, male: ≥ 80 cm

• HDL-c

< 16 years: 40 mg/dL

≥ 16 years, male: < 40 mg/dL

≥ 16 years, female: < 50 mg/dL

• Triglycerides: ≥ 150 mg/dL

• Glucose: ≥ 100 mg/dL

• Systolic blood pressure ≥ 130 mmHg or diastolic blood pressure ≥ 85 mmHg

Adolescents who had any disability that would prevent weight, height and WC from being evaluated were excluded from this study. Pregnant women were also excluded.

Prevalences of MS were estimated with their respective 95% confidence intervals (95%CI) in the strata corresponding to the Brazilian macro-regions, according to sex and age groups, from 12 to 14 and 15 to 17 years of age. The complex sample design was taken into account, with weighting included due to the different probabilities of cluster selection and subsequent calibration by age and sex, from which the population estimates were obtained^a^.The prevalences of MS were also estimated by the schools’ status (public or private), in obese and nonobese adolescents, as were the risk factors that make up the IDF criterion for MS. Stata^b^ software 14 version was used for data analysis.

ERICA was approved by the Committee for Ethics in Research (CEP) of the Institute of Collective Health Studies, Universidade Federal do Rio de Janeiro (Process 45/2008) and by a CEP of each unit of the Federation. The adolescents who participated in this study agreed in writing to participate and their legal guardians signed an informed consent form.

## RESULTS

**Figure 1:**
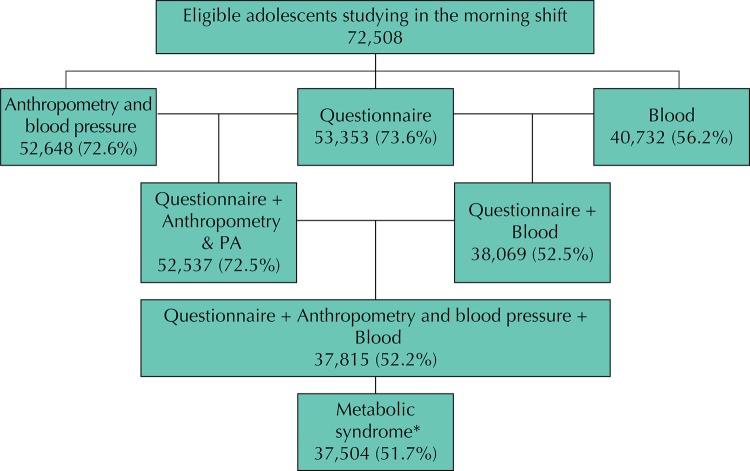
Flowchart of the participants assessed regarding the prevalence of metabolic syndrome. ERICA, Brazil, 2013-2014.

Figure 1 presents a flow chart of the participants. We evaluated 37,504 adolescents, of which 60.0% were female, 54.3% were aged between 15 to 17 years and 73.3% studied in public schools.

* 311 adolescents did not have results from all the tests required for diagnosing metabolic syndrome.

The prevalence of MS in schools in Brazilian cities with more than 100,000 inhabitants, for students studying in the morning, is presented in Table 1. Regarding the prevalence of MS by sex and age group, small variations occurred in the prevalence with an overlapping of the 95% confidence interval (Table 2). In relation to age group, in most regions the prevalence was higher in adolescents aged between 15 and 17 years. However, in the South region, the prevalence of MS in adolescents aged between 12 and 17 years was greater than in those between 15 and 17 years, albeit with an overlapping of the confidence intervals; for male adolescents the opposite occurred together with statistical significance. In the Midwest region, younger male adolescents showed greater MS prevalence than the older age group, from 15 to 17 years of age.

**Table 1:** Prevalences (%) and 95%CI of metabolic syndrome, sample size and estimated population in the set of cities with more than 100,000 inhabitants in Brazil, according to sex and age group. ERICA, 2013-2014.

Characteristic	Age group	Sample	Population	%	95%CI
Female	12-14	10,134	1,536,757	2.5	1.7-3.6
	15-17	12,365	1,788,116	2.1	1.5-2.7
	12-17	22,499	3,324,873	2.2	1.8-2.8
Male	12-14	7,016	1,552,255	2.5	2.0-3.2
	15-17	7,990	1,751,833	3.3	2.5-4.2
	12-17	15,006	3,304,088	2.9	2.5-3.4
Brazil	12-14	17,150	3,089,012	2.5	2.0-3.0
	15-17	20.355	3,539,949	2.7	2.2-3.2
	12-17	37,504	6,628,961	2.6	2.3-2.9

**Table 2:** Prevalences (%) and 95%CI of metabolic syndrome, sample size and estimated population in cities with more than 100,000 inhabitants, according to Brazilian regions, sex and age group. ERICA, 2013-2014.

Regions/Sex	Age group	Sample	Population	%	95%CI
North		7,233	456,416	2.3	2.0-2.8
Female	12-14	1,979	126,869	2.0	1.3-3.0
	15-17	2,279	100,881	2.5	1.8-3.4
Male	12-14	1,458	127,856	1.6	0.9-2.8
	15-17	1,517	100,810	3.5	2.6-4.7
Northeast		11,661	1,337,676	2.7	2.3-3.1
Female	12-14	3,314	343,871	2.2	1.3-4.0
	15-17	3,722	325,047	2.3	1.7-3.3
Male	12-14	2,160	350,923	2.7	1.8-4.1
	15-17	2,465	317,835	3.4	2.6-4.4
Midwest		5,441	525,340	2.2	1.7-3.0
Female	12-14	1,543	123,669	1.9	1.1-3.1
	15-17	1,802	140,324	2.1	1.4-3.3
Male	12-14	1,060	124,867	3.2	1.8-5.4
	15-17	1,056	136,480	1.8	1.1-3.0
Southeast		8,460	3,488,424	2.4	1.9-3.0
Female	12-14	2,141	755,994	1.9	1.0-3.7
	15-17	3,024	995,770	1.9	1.1-3.1
Male	12-14	1,459	760,343	2.7	1.8-4.0
	15-17	1,836	976,317	3.1	2.0-4.8
South		4,690	821,105	3.5	2.6-4.9
Female	12-14	1,157	186,354	5.7	2.5-12.7
	15-17	1,538	226,094	2.1	1.0-4.3
Male	12-14	879	188,266	1.6	0.9-2.8
	15-17	1,116	220,391	4.8	3.3-6.9

**Figure 2:**
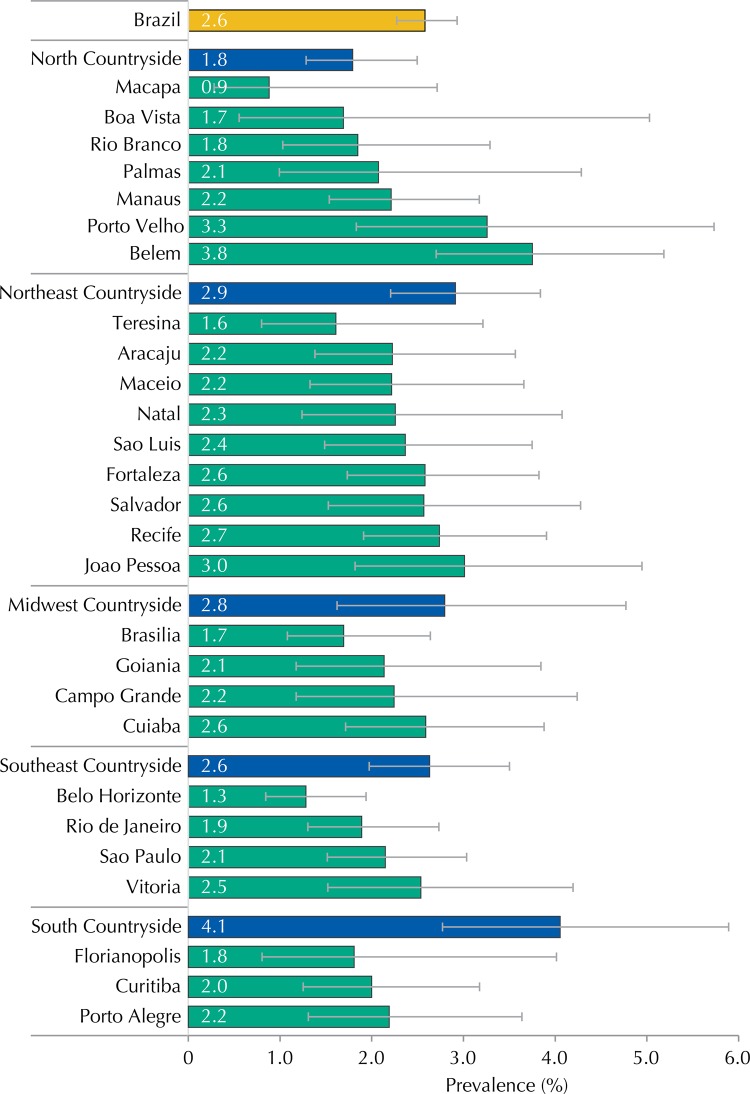
Prevalences (%) and 95%CI of metabolic syndrome in adolescents according to strata of the capital and countryside regions. ERICA, Brazil, 2013-2014.

The Brazilian state capital that presented the highest MS prevalence was Belem (3.8% [95%CI 2.7-5.2]), with the smallest being Macapa (0.9% [95%CI 0.3-2.7]), which are both in the North region and had the largest variations between them (Figure 2). However, when all the strata from the sample are considered, the greatest MS prevalence was observed in the countryside cities of the South macro-region (4.1% [95%CI 2.8-5.9]).

The prevalence of MS was 2.8% in public schools (95%CI 2.4-3.2) and 1.9% in private schools (95%CI 1.4-2.4). In public schools, the prevalence of MS was 3.1% in male adolescents (95%CI 2.5-3.7) and 2.6% in the females (95%CI 2.0-3.3). In private schools, the prevalence was lower in both male (2.5% [95%CI 1.9-3.3]) and female adolescents (1.3% [95%CI 0.9-1.9]).

The prevalences of MS in adolescents with adequate nutritional status were less than 0.2%, both in male (0.008% [95%CI 0.003-0.03]) and female (0.1% [95%CI 0.05-0.4]) adolescents. In the North, Northeast and Southeast regions the prevalence of MS in this group of adolescents was 0.1%, and lower still in the Midwest and South regions.

Table 3 shows the prevalence of MS, by region and sex, in overweight or obese adolescents. The prevalence of MS was more than six times greater in obese adolescents than in overweight adolescents. This proportion was always greater in male adolescents, with the exception being in the South region, in which the highest ratio among the regions was also observed. The prevalences of MS and the 95%CI by nutritional status, according to sex or age group in Brazil and the macro-regions, can be seen in Table 4.

**Table 3:** Prevalences (%) and 95%CI of metabolic syndrome in overweight or obese adolescents, according to sex and macro-region. ERICA, Brazil, 2013-2014.

Regions/Sex	Overweight		Obesity	
	
	%	95%CI	%	95%CI
Brazil	3.3	2.4-4.5	21.3	18.5-24.5
Female	4.0	2.6-6.0	17.5	13.7-21.9
Male	2.6	1.6-4.1	24.5	20.1-29.6
North	3.3	2.4-4.4	24.7	20.5-29.5
Female	5.1	3.6-7.1	22.4	16.6-29.5
Male	1.3	0.7-2.6	26.3	20.1-33.6
Northeast	3.3	2.1-5.3	21.7	16.7-27.8
Female	3.5	2.2-5.6	20.1	12.2-31.2
Male	3.1	1.7-5.7	22.8	17.9-28.6
Midwest	2.1	1.2-3.0	21.7	16.1-27.9
Female	3.1	1.9-5.1	23.9	16.2-33.8
Male	1.2	0.5-2.4	20.5	14.8-27.7
Southeast	3.9	2.4-6.2	18.9	14.5-24.2
Female	4.8	2.5-9.1	11.3	7.8-16.0
Male	3.0	1.4-6.1	26.1	17.9-36.4
South	1.6	0.9-3.0	27.3	20.7-35.2
Female	1.8	0.8-4.0	31.8	19.4-47.5
Male	1.4	0.6-3.4	23.6	16.5-32.6

**Table 4:** Prevalences (%) and 95%CI of metabolic syndrome in adolescents by sex, age and Brazilian regions according to nutritional status. ERICA, 2013-2014.

Characteristic	Nutritional status	%	95%CI
Brazil	Eutrophic	0.1	0.02-0.2
	Overweight	3.3	2.4-4.5
	Obese	21.3	18.5-24.5
Female	Eutrophic	0.1	0.03-0.4
	Overweight	4.0	2.6-6.0
	Obese	17.5	13.7-21.9
Male	Eutrophic	0.008	0.003-0.03
	Overweight	2.6	1.6-4.1
	Obese	24.5	20.1-29.6
12-14 years	Eutrophic	0.01	0.006-0.04
	Overweight	1.7	0.7-3.8
	Obese	19.6	15.8-24.1
15-17 years	Eutrophic	0.1	0.04-0.4
	Overweight	4.9	3.6-6.6
	Obese	23.5	19.1-28.6
North	Eutrophic	0.1	0.02-0.2
	Overweight	3.3	2.4-4.4
	Obese	24.7	20.5-29.5
Northeast	Eutrophic	0.1	0.03-0.2
	Overweight	3.3	2.1-5.3
	Obese	21.7	16.89-27.5
Midwest	Eutrophic	0.02	0.01-0.1
	Overweight	1.6	0.9-3.0
	Obese	27.3	20.7-35.2
Southeast	Eutrophic	0.02	0.0-0.1
	Overweight	2.1	1.4-3.2
	Obese	21.7	16.7-27.8
South	Eutrophic	0.1	0.01-0.4
	Overweight	3.9	2.5-6.2
	Obese	18.9	14.5-24.2

**Figure 3:**
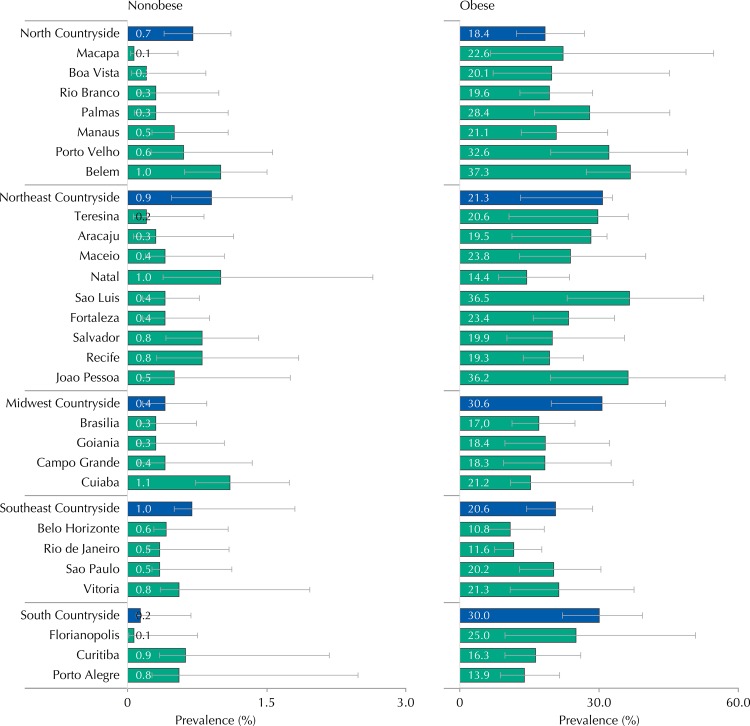
Prevalences (%) and 95%CI of metabolic syndrome in obese and nonobese adolescents according to strata of the capital and countryside regions. ERICA, Brazil, 2013-2014.

Figure 3 shows that, in all strata from the sample, the prevalence of MS was higher in obese adolescents than in nonobese ones. The prevalence of MS and confidence intervals in the strata of the sample can be seen in Table 5.

**Table 5:** Prevalences (%) and 95%CI of metabolic syndrome in adolescents by capital and strata of the countryside regions. ERICA, Brazil, 2013-2014.

Strata	Total		Nonobese		Obese	
		
	%	95%CI	%	95%CI	%	95%CI
North Countryside	1.8	1.3-2.5	0.7	0.4-1.1	18.4	12.2-26.9
Porto Velho	3.3	1.8-5.7	0.6	0.3-1.6	32.6	19.6-49.0
Rio Branco	1.9	1.0-3.3	0.3	0.1-1.0	19.6	12.9-28.6
Manaus	2.2	1.5-3.2	0.5	0.3-1.1	21.1	13.2-31.8
Boa Vista	1.7	0.6-5.0	0.2	0-0.8	20.1	7.2-45.1
Belem	3.8	2.7-5.2	1.0	0.6-1.5	37.3	27.2-48.7
Macapa	0.9	0.3-2.7	0.1	0-0.5	22.6	6.6-54.6
Palmas	2.1	1.0-4.3	0.3	0.1-1.1	28.4	16.1-45.2
Northeast Countryside	2.9	2.2-3.8	0.9	0.5-1.8	21.3	13.0-32.9
Sao Luis	2.4	1.5-3.7	0.4	0.2-0.8	36.5	23.1-52.5
Teresina	1.6	0.8-3.2	0.2	0.1-0.8	20.6	10.6-36.3
Fortaleza	2.6	1.7-3.8	0.4	0.2-0.9	23.4	15.8-33.3
Natal	2.3	1.2-4.1	1.0	0.4-2.6	14.4	8.3-23.6
Joao Pessoa	3.0	1.8-4.9	0.5	0.1-1.8	36.2	19.5-57.1
Recife	2.7	1.9-3.9	0.8	0.3-1.8	19.3	13.7-26.6
Maceio	2.2	1.3-3.7	0.4	0.2-1.0	23.8	12.8-40.0
Aracaju	2.2	1.4-3.6	0.3	0.1-1.1	19.5	11.2-31.7
Salvador	2.6	1.5-4.3	0.8	0.4-1.4	19.9	10.1-35.5
Midwest Countryside	2.8	1.6-4.8	0.4	0.2-0.9	30.6	19.7-44.3
Campo Grande	2.2	1.2-4.2	0.4	0.1-1.3	18.3	9.4-32.6
Cuiaba	2.6	1.7-3.9	1.1	0.7-1.7	21.2	10.9-37.3
Goiania	2.1	1.2-3.8	0.3	0.1-1.0	18.4	9.7-32.2
Brasilia	1.7	1.1-2.6	0.3	0.1-0.7	17.0	11.2-24.8
Southeast Countryside	2.6	2.0-3.5	1.0	0.5-1.8	20.6	14.4-28.6
Belo Horizonte	1.3	0.8-1.9	0.6	0.3-1.1	10.8	6.2-18.2
Vitoria	2.5	1.5-4.2	0.8	0.3-2.0	21.3	10.8-37.5
Rio de Janeiro	1.9	1.3-2.7	0.5	0.2-1.1	11.6	7.5-17.7
Sao Paulo	2.1	1.5-3.0	0.5	0.3-1.1	20.2	12.9-30.4
South Countryside	4.1	2.8-5.9	0.2	0.1-0.7	30.0	22.1-39.3
Curitiba	2.0	1.3-3.2	0.9	0.3-2.2	16.3	9.7-26.0
Florianopolis	1.8	0.8-4.0	0.1	0.0-0.7	25.0	9.7-50.7
Porto Alegre	2.2	1.3-3.6	0.8	0.3-2.5	13.9	8.7-21.5

Regarding the composition of the MS, which according to the adopted criteria must include the presence of enlarged WC, 82.3% (95%CI 79.2-85.0) of the adolescents with MS were observed to present three components, 15.6% (95%CI 13.0-18.6) had four components, and 2.1% (95%CI 1.0-4.5) had five components.

The most frequently observed MS component combinations were: enlarged WC, low HDL-c and high blood pressure at 33.4%; enlarged WC, low HDL-c and high triglycerides at 31.8%; and enlarged WC, low HDL-c and high triglycerides and blood pressure at 9.5%. These combinations of factors represented about 3/4 of all possible combinations.

Table 6 describes the prevalence of MS components throughout the sample, the prevalence of MS in those with each of the components and the prevalence of each component in adolescents with MS. About 1/3 of the adolescents had low HDL-c, which was reflected in the high prevalence of this component among adolescents with MS; however, less than 10.0% of adolescents with this variation had MS. Concerning high triglycerides, despite having been presented at a low prevalence compared to the other components in the total sample, they were present in almost half of all the adolescents with MS; and the prevalence of MS in those with this variation was high (26.8%), even greater than in those with the required component, i.e., enlarged WC (20.5%). High glucose levels, which had the lowest prevalence of all the components in the total sample, was associated with MS prevalence of around two times greater than in the presence of low HDL-c. Nearly 20.0% of adolescents with high blood pressure, according to the IDF criteria, were classified as having MS; and among the adolescents with MS, just over half had high blood pressure (Table 6).

**Table 6:** Prevalence (%) and 95%CI of MS components in the population, of MS in adolescents with particular components, and of components in adolescents with MS. ERICA, Brazil, 2013-2014.

MS components	Prevalence of the component		Prevalence of MS in adolescents with the component		Prevalence of the component in adolescents with MS
		
	%	95%CI	%	95%CI	%
WC^a^	12.6	11.6-13.7	20.5	18.2-22.9	100
HDL-c^b^	32.7	30.3-35.2	7.2	6.3-8.2	90.7
Triglycerides^a^	4.6	4.1-5.1	26.8	22.4-31.7	47.6
Glucose^a^	4.1	3.5-4.8	15.0	10.8-20.4	20.6
Blood pressure^a^	8.2	7.6-8.9	18.7	15.6-22.1	57.6

MS: metabolic syndrome; WC: waist circumference; HDL-c: HDL-cholesterol

^a^ High.

^b^ Low.

## DISCUSSION

This was the first study performed with a representative sample of the Brazilian population, in this age group, in which important information is shown regarding the prevalence of MS. The observed prevalences varied according to the macro-region, sex and age without presenting any specific single pattern. The prevalence of MS in the South region was greater than in the other regions, mostly due to the prevalence observed in the cities with more than 100,000 inhabitants in the countryside of this macro-region. This fact can be attributed to different eating habits and lifestyles in relation to the macro-regions, since these are the main factors in the genesis of obesity, which is a central component in MS diagnosis.

We observed higher prevalence of MS in adolescents from public schools, which indicates a possible association between socioeconomic factors and MS. Analyzing the characteristics that may vary according to socioeconomic status, such as parenting, eating habits or physical activities, can help in the understanding of these relationships. Some studies observed an opposite relationship to what was found in ERICA, i.e., a greater prevalence of MS in higher income individuals^5^ ^,^ ^7^ ^,^ ^8^.

More than 40 MS definitions for children and adolescents exist^11^. The MS definition proposed by the IDF puts forward prevalences that tend to be lower than those estimated with other definitions of frequent use. Some studies with smaller proportions than those with ERICA, such as Alvarez et al.^1^, who evaluated 577 adolescents from public and private schools in the Brazilian city of Niteroi, RJ, Southeastern Brazil, observed an MS prevalence of 1.6% (95%CI 0.6-3.9) using the IDF definition and 6.0% (95%CI 3.0-7.8) using the NCEP-ATPIII criteria. This difference may have been due to the required presence of enlarged WC in the IDF definition.

No consensus exists regarding the most suitable criteria, and adolescents represent a fraction of the population, whose main characteristic is defined by transformation. Therefore, using a more specific definition (not labeling those with a low probability of having this syndrome or their false-positives as MS patients) seems more appropriate than using a more sensitive criterion.

During ERICA, the prevalence of MS among the obese only differed in terms of sex in the North and Southeast regions. In the North, it was higher among female overweight adolescents; and in the Southeast, it was obese males. Most research projects report a higher prevalence in male adolescents^21^ ^,^ ^23^.

While using data on adolescents from the National Health and Nutrition Examination Survey (NHANES), Laurson et al.^13^ noted, in accordance with the NCEP-ATPIII criteria, an 0.8% prevalence of MS in 1,785 male adolescents with adequate nutritional status (95%CI 0.1-1.5), 6.8% in overweight individuals (95%CI 1.2-12.4) and 35.4% in the obese (95%CI 27.1-43.7). In 1,600 female adolescents, we observed MS prevalence of 1.7% for individuals who were neither overweight or obese (95%CI 0.1-3.3), 9.2% for overweight females (95%CI 4.4-14.0) and 24.6% for the obese (95%CI 17.2-31.9). In this study, the prevalence of MS was lesser in both male and female adolescents, regardless of nutritional status, when compared to the prevalence found in the study by Laurson et al., albeit with different criteria employed. Nasreddine et al.^16^, while using the IDF criteria, observed a prevalence of 21.2% in obese Lebanese adolescents, 3.8% in the overweight and 1.2% in individuals who were neither overweight or obese, which are similar results to those observed during ERICA.

In this study, the prevalence of the MS components, observed in adolescents with MS, in descending order, were: enlarged WC, low HDL-c, high triglycerides, blood pressure and glucose levels. Nasreddine et al.^16^ observed a similar descending order. However, analyzing the contribution of each MS component enables the visualization of the importance of the individualized model. Despite the prevalence of low HDL-c having been singularly the highest, the prevalence of MS was higher in adolescents with high triglycerides or high blood pressure. In other words, according to the IDF criteria, the triglycerides represented a relevant component.

MS is directly associated with insulin resistance and it is this that enables the promotion and explanation of the emergence, prevalence and magnitude of each of the MS components^3^ ^,^ ^14^ ^,^ ^19^. The presence of only one of the MS components supports non-medicinal interventions, such as promoting healthy lifestyle habits, in addition to follow-up at health services. Despite the MS prevalences having been low during ERICA, we observed a strong association with nutritional status, which indicates that preventing obesity can have a significant impact on reducing MS prevalence as well as its subsequent cardiovascular complications during adult life.

## Footnotes

a Instituto Brasileiro de Geografia e Estatística. Projeção da população do Brasil por sexo e idade – 1980-2050: revisão 2008. Rio de Janeiro (RJ): IBGE; 2008 [cited 2015 Sept]. (Estudos e pesquisas, 24). Available from: http://www.ibge.gov.br/home/estatistica/populacao/projecao_da_populacao/2008/projecao.pdf

b Stata Corp. Stata statistical software: release 14. College Station, Texas; 2015.
