# Erratum

**DOI:** 10.1590/S01518-8787.2016050006701err

**Published:** 2016-11-29

In article: “ERICA: prevalence of metabolic syndrome in Brazilian adolescents”, DOI:10.1590/S01518-8787.2016050006701, published in the journal “Revista de Saúde Pública”, volume 50 (2016), supplement ERICA, article 11s.

**Where you read:**

How to cite: **Kyscgubur NCC** , Bloch KV, Szklo M, Klein CH, Barufaldi LA, Abreu GA et al. ERICA: prevalência de síndrome metabólica em adolescentes brasileiros. Rev Saude Publica. 2016;50(supl 1):11s.

**You should read:**

How to cite: **Kyscgubur MCC** , Bloch KV, Szklo M, Klein CH, Barufaldi LA, Abreu GA et al. ERICA: prevalência de síndrome metabólica em adolescentes brasileiros. Rev Saude Publica. 2016;50(supl 1):11s.

