# Supplementary material for: ERICA: prevalence of metabolic syndrome in Brazilian adolescents
Source: Rev Saude Publica. 2016 Feb 2;50(Suppl 1):11s. doi: 10.1590/S01518-8787.2016050006701 (PMC4767042; doi:10.1590/S01518-8787.2016050006701)
Supplement: Translation

# Translation: ERICA: prevalência de síndrome metabólica em adolescentes brasileiros

**Authors:** Maria Cristina C Kuschnir, Katia Vergetti Bloch, Moyses Szklo, Carlos Henrique Klein, Laura Augusta Barufaldi, Gabriela de Azevedo Abreu, Beatriz Schaan, Gloria Valeria da Veiga, Thiago Luiz Nogueira da Silva, Maurício Teixeira Leite de Vasconcellos, e demais autores

**Affiliations:** I Núcleo de Estudos da Saúde do Adolescente. Faculdade de Ciências Médicas. Universidade do Estado do Rio de Janeiro. Rio de Janeiro, RJ, Brasil; II Instituto de Estudos em Saúde Coletiva. Universidade Federal do Rio de Janeiro. Rio de Janeiro, RJ, Brasil; III Escola Nacional de Saúde Pública. Fundação Oswaldo Cruz. Rio de Janeiro, RJ, Brasil; IV Programa de Pós-Graduação em Saúde Coletiva. Instituto de Medicina Social. Universidade do Estado do Rio de Janeiro. Rio de Janeiro, RJ, Brasil; V Hospital de Clínicas de Porto Alegre. Universidade Federal do Rio Grande do Sul. Porto Alegre, RS, Brasil; VI Instituto de Nutrição Josué de Castro. Universidade Federal do Rio de Janeiro. Rio de Janeiro, RJ, Brasil; VII Escola Nacional de Ciências Estatísticas. Fundação Instituto Brasileiro de Geografia e Estatística. Rio de Janeiro, RJ, Brasil

**Keywords:** Adolescente, Síndrome Metabólica, epidemiologia, Fatores de Risco, Doenças Cardiovasculares, Estudos Transversais

## Abstract

**OBJETIVO:**

Determinar a prevalência de síndrome metabólica e de seus componentes em adolescentes brasileiros.

**MÉTODOS:**

Foram avaliados 37.504 adolescentes, participantes do Estudo de Riscos Cardiovasculares em Adolescentes (ERICA), estudo transversal de âmbito nacional e de base escolar. Os adolescentes, de 12 a 17 anos de idade, residiam em municípios com mais de 100 mil habitantes. A amostra foi estratificada e conglomerada em escolas e turmas. Os critérios da *International Diabetes Federation* foram utilizados para definir síndrome metabólica. Prevalências de síndrome metabólica foram estimadas segundo sexo, faixa etária, tipo de escola e estado nutricional.

**RESULTADOS:**

Dos 37.504 adolescentes, 50,2% eram do sexo feminino; 54,3% tinham de 15 a 17 anos e 73,3% estudavam em escolas públicas. A prevalência nacional de síndrome metabólica foi 2,6% (IC95% 2,3-2,9), discretamente maior no sexo masculino e naqueles de 15 a 17 anos na maioria das macrorregiões. A prevalência foi a maior nos residentes na macrorregião Sul, nas adolescentes mais jovens e nos adolescentes mais velhos. A prevalência foi maior nas escolas públicas (2,8% [IC95% 2,4-3,2]) que nas escolas privadas (1,9% [IC95% 1,4-2,4]) e nos adolescentes obesos em comparação aos não obesos. As combinações de componentes mais frequentes, respondendo por 3/4 das combinações, foram: circunferência de cintura elevada, HDL-colesterol baixo e pressão arterial elevada, seguida de circunferência da cintura elevada, lipoproteína de alta densidade (HDL-c) baixo e triglicerídeos elevados, e wpor circunferência da cintura elevada, HDL baixa e triacilgliceróis e pressão arterial elevados. A HDL baixa foi o segundo componente mais frequente, mas a maior prevalência de síndrome metabólica (26,8%) foi observada na presença de triglicerídeos elevado.

**CONCLUSÕES:**

O ERICA é o primeiro estudo nacional a apresentar prevalências de síndrome metabólica e descrever a participação dos seus componentes. Apesar de a prevalência da síndrome metabólica ter sido baixa, as altas prevalências de alguns componentes e de participação de outros na composição da síndrome torna importante o diagnóstico precoce de tais alterações, mesmo que não agrupadas na síndrome metabólica.

## INTRODUÇÃO

O aumento na prevalência de excesso de peso e obesidade em crianças e adolescentes, observado em diversos países^18^ ^,^ incluindo o Brasil^6^ ^,^ ^15^, gera grave problema de saúde pública, pelos diversos riscos à saúde que a obesidade pode ocasionar, tanto na adolescência, quanto na vida adulta. Entre os fatores de risco cardiovascular já reconhecidos estão aqueles que compõem a chamada síndrome metabólica (SM). Estão associados à SM a circunferência da cintura (CC) elevada, o colesterol ligado à lipoproteína de alta densidade – *high density lipoprotein* (HDL-c) baixo e a pressão arterial sistêmica, os triglicerídeos, e a glicemia elevados. O diagnóstico de SM é positivo se o indivíduo apresentar pelo menos três dessas condições, que se relacionam e têm como um dos desfechos desfavoráveis o desenvolvimento da doença cardiovascular^24^.

Existem várias propostas para definição de SM em crianças e adolescentes. No entanto, não existe consenso sobre quais seriam seus componentes e respectivos pontos de corte, já definidos para adultos. Em 2004, Ferranti et al.^10^ propuseram uma adaptação dos critérios criados pelo *National Cholesterol Education Program Adult Treatment Panel* (NCEP-ATPIII). De acordo com esta definição de SM, o ponto de corte da CC é o percentil 70^10^.

A *International Diabetes Federation* (IDF) estabeleceu um novo conceito de SM para crianças e adolescentes em 2007, que considera a medida aumentada da CC como componente principal e sua presença imprescindível para definição da SM. Assim, o diagnóstico da SM passou a ser realizado em crianças com 10 anos ou mais, caso a CC fosse maior ou igual ao percentil 90 da curva elaborada por Fernandez et al.^9^, concomitantemente à presença de dois ou mais dos critérios clínicos ou laboratoriais (HDL-c baixo e pressão arterial, triglicerídeos e glicose elevados). Em adolescentes com mais de 16 anos são usados os critérios da IDF para adultos^25^. Giannini et al.^12^ em 2014 observaram que, comparados aos critérios do NCEP-ATPIII, a utilização dos critérios da IDF resultou em prevalências menores em uma amostra de adolescentes com excesso de peso. Tavares et al.^22^, em revisão sistemática de 15 artigos realizados com adolescentes brasileiros, 11 dos quais na região Sudeste, observaram variação entre 0% e 42,0% na prevalência da SM, dependendo do critério de diagnóstico utilizado, sendo mais acentuada nos indivíduos com excesso de peso.

O objetivo deste estudo foi determinar a prevalência da SM em adolescentes brasileiros.

## MÉTODOS

O ERICA foi planejado para estimar a prevalência de fatores de risco cardiovascular que fazem parte da SM em amostra representativa de adolescentes de 12 a 17 anos. Foram selecionados adolescentes que frequentavam o sétimo, oitavo e nono ano do ensino fundamental ou o primeiro, segundo ou terceiro ano do ensino médio de escolas públicas ou privadas, em turmas da manhã, em municípios brasileiros com mais de 100 mil habitantes. A escolha desses anos escolares teve como objetivo incluir o maior número possível de adolescentes de 12 a 17 anos de idade, uma vez que não havia base de informação de unidades individuais de adolescentes para seleção amostral, mas apenas de combinações de turmas e anos em escolas.

A amostra foi estratificada em 32 estratos, constituídos pelos 27 municípios de capital das unidades da federação e por cinco conjuntos dos demais municípios, do interior, de mais de 100 mil habitantes, de cada uma das cinco macrorregiões do País. Assim, a amostra tem representatividade para o conjunto de municípios de médio e grande porte no nível nacional, regional e para as capitais e o Distrito Federal. Foram selecionados conglomerados em três níveis: escolas, combinações de ano e turno, e turmas. Os pesos amostrais foram calculados pelo produto dos inversos das probabilidades de inclusão em cada estágio de seleção da amostra e foram calibrados considerando as estimativas de população de adolescentes matriculados em escolas localizadas nos estratos geográficos considerados, por sexo e idade. A análise foi ajustada para considerar o desenho amostral, com o uso de rotinas estatísticas para amostragem complexa que consideram as fontes de variabilidade e a calibração com estimativas populacionais.

O cálculo do tamanho amostral considerou a prevalência esperada de SM em adolescentes de 4,0%^6^, com erro máximo de 0,9% e com nível de confiança de 95%, além de um efeito de aglomeração de 2,97. A descrição pormenorizada do processo de amostragem encontra-se em Vasconcellos et al.^23^

Para a estimativa da prevalência de SM foram realizadas análises bioquímicas no plasma. A coleta de sangue exigia jejum de 12 horas, e, portanto, só foi realizada nos estudantes das turmas da manhã selecionadas na amostra ERICA. Assim, a inferência dos resultados relativos à SM só pode ser feita para estudantes que estudam no turno da manhã.

As variáveis sexo e idade foram obtidas por meio de questionário autopreenchido em coletor de dados eletrônico (PDA) que continha também outras questões não utilizadas nesta análise. A idade foi confirmada com o auxílio dos registros escolares. Foram aferidas, por pesquisadores treinados, as seguintes variáveis antropométricas: CC, medida da circunferência do braço direito (para a escolha do manguito apropriado para medida da pressão arterial), peso e estatura. Essas medidas foram monitoradas ao longo da coleta por meio de controle de qualidade que verificou seus limites e distribuição de dígitos terminais.

O peso foi avaliado em balança eletrônica da marca Líder^®^ modelo P200M com capacidade de até 200 kg e variação de 50 g. A estatura foi aferida duas vezes, utilizando-se estadiômetro portátil da marca Altura Exata^®^ com variação de 0,1 cm, sendo considerada a média dos dois valores obtidos. As medidas de peso e altura foram utilizadas para a classificação do estado nutricional a partir do cálculo do índice de massa corporal (IMC = peso/estatura^2^). A classificação do estado nutricional utilizou as curvas de IMC propostas pela Organização Mundial da Saúde^17^ (2007) específicas por idade e sexo. Foram considerados como tendo estado nutricional adequados os adolescentes com escore +1 > Z ≥ -2, com sobrepeso os adolescentes com escore +2 > Z ≥ +1, e com obesidade aqueles com escore Z ≥ +2.

A CC foi medida com fita métrica inextensível da marca Sanny^®^, com variação de 0,1 cm, no ponto médio entre a curvatura inferior da última costela fixa e a curvatura superior da crista ilíaca com o adolescente em pé, braços ao longo do corpo, pés unidos e abdômen relaxado.

A pressão arterial foi aferida com o aparelho Omron^®^ 705-IT, validado para uso em adolescentes^21^. Das três medidas de pressão arterial realizadas, foi utilizada na classificação de hipertensão a média entre as duas últimas. A pressão arterial sistêmica foi considerada elevada se a pressão arterial sistólica fosse maior ou igual a 130 mmHg e a diastólica, maior ou igual a 85 mmHg.

As análises em amostras de sangue dos parâmetros bioquímicos que compõem a SM (HDL-c, triglicerídeos e glicose) foram realizadas em um único laboratório que seguiu as normas de qualidade vigentes e exigidas para sua qualificação. A glicemia de jejum foi avaliada pelo método enzimático GOD-PAP no equipamento Roche modular analítico. Os valores de 100 mg/dL ou mais foram considerados como discriminantes de glicemia elevada^2^. Triglicerídeos e HDL-c foram analisados pelo método enzimático calorimétrico no equipamento Roche modular analítico. Foram utilizados os valores de referência divulgados na I Diretriz de Prevenção da Aterosclerose na Infância e na Adolescência^20^. A descrição completa dos métodos do ERICA encontra-se em Bloch et al.^4^

A classificação positiva da SM foi realizada pela presença de pelo menos três dos componentes abaixo, com presença obrigatória da CC elevada, de acordo com o critério da *International Diabetes Federation* ^2^:

• CC

< 16 anos: ≥ Percentil 90

≥ 16 anos, sexo masculino: ≥ 90 cm

≥ 16 anos, sexo feminino: ≥ 80 cm

• HDL-c

< 16 anos: 40 mg/dL

≥ 16 anos, sexo masculino: < 40 mg/dL

≥ 16 anos, sexo feminino: < 50 mg/dL

• Triglicerídeos: ≥ 150 mg/dL

• Glicose: ≥ 100 mg/dL

• Pressão arterial sistólica ≥ 130 mmHg ou diastólica ≥ 85 mmHg

Neste estudo foram excluídos os adolescentes que apresentaram qualquer deficiência física que impedisse a avaliação de peso, estatura e CC. Foram também excluídas as grávidas.

As prevalências de SM foram estimadas com os respectivos intervalos de confiança de 95% (IC95%) nos estratos correspondentes às macrorregiões brasileiras, segundo sexo e grupos etários, de 12 a 14 e de 15 a 17 anos. Foi levado em consideração o delineamento de amostra complexa, com ponderação devido às diferentes probabilidades de seleção dos conglomerados e posterior calibração pelos domínios de idade e sexo, para os quais foram obtidas estimativas populacionais^a^ ^.^ Foram estimadas também as prevalências de SM por natureza da escola (pública ou privada), em adolescentes obesos e não obesos, assim como dos fatores de risco que compõem o critério da IDF para SM. O programa Stata^b^, versão 14, foi utilizado para a análise dos dados.

O ERICA foi aprovado pelo Comitê de Ética em Pesquisa (CEP) do Instituto de Estudos em Saúde Coletiva, da Universidade Federal do Rio de Janeiro (Processo 45/2008) e por um CEP de cada unidade da Federação. Participaram desse estudo os adolescentes que concordaram, por escrito, em participar e cujos responsáveis assinaram o termo de consentimento livre e esclarecido.

## RESULTADOS

A Figura 1 apresenta o fluxograma dos participantes. Foram avaliados 37.504 adolescentes, dos quais 60,0% era do sexo feminino, 54,3% tinha de 15 a 17 anos e 73,3% estudava em escolas públicas.

**Figura 1:** Fluxograma dos participantes avaliados quanto à prevalência de síndrome metabólica. ERICA, Brasil, 2013-2014. * 311 adolescentes não tiveram resultados de todos os exames necessários para diagnóstico da síndrome metabólica.

As prevalências de SM em escolares de municípios com mais de 100 mil habitantes no Brasil, que estudam no turno da manhã, são apresentadas na Tabela 1. Em relação à prevalência de SM por sexo e faixa etária, observaram-se pequenas variações na prevalência com sobreposição dos IC95% (Tabela 2). Em relação à faixa etária, na maioria das regiões a prevalência foi maior nos adolescentes com 15 a 17 anos. Entretanto, na região Sul, a prevalência de SM nas adolescentes de 12 a 17 anos foi maior que naquelas de 15 a 17 anos, ainda que com superposição dos intervalos de confiança; nos adolescentes do sexo masculino, ocorreu o contrário, e com significância estatística. Na região Centro-Oeste, foram os adolescentes do sexo masculino mais jovens que apresentaram prevalência de SM maior do que os mais velhos, de 15 a 17 anos.

**Tabela 1:** Prevalências (%) e IC95% de síndrome metabólica, tamanho da amostra e população estimada no conjunto de municípios de mais de 100 mil habitantes no Brasil, segundo sexo e faixa etária. ERICA, 2013-2014.

Característica	Faixa etária	Amostra	População	%	IC95%
Feminino	12-14	10.134	1.536.757	2,5	1,7-3,6
	15-17	12.365	1.788.116	2,1	1,5-2,7
	12-17	22.499	3.324.873	2,2	1,8-2,8
Masculino	12-14	7.016	1.552.255	2,5	2,0-3,2
	15-17	7.990	1.751.833	3,3	2,5-4,2
	12-17	15.006	3.304.088	2,9	2,5-3,4
Brasil	12-14	17.150	3.089.012	2,5	2,0-3,0
	15-17	20.355	3.539.949	2,7	2,2-3,2
	12-17	37.504	6.628.961	2,6	2,3-2,9

**Tabela 2:** Prevalências (%) e IC95% de síndrome metabólica, tamanho da amostra e população estimada nos municípios com mais de 100 mil habitantes, segundo regiões brasileiras, sexo e faixa etária. ERICA, 2013-2014.

Regiões/Sexo	Faixa etária	Amostra	População	%	IC95%
Norte		7.233	456.416	2,3	2,0-2,8
Feminino	12-14	1.979	126.869	2,0	1,3-3,0
	15-17	2.279	100.881	2,5	1,8-3,4
Masculino	12-14	1.458	127.856	1,6	0,9-2,8
	15-17	1.517	100.810	3,5	2,6-4,7
Nordeste		11.661	1.337.676	2,7	2,3-3,1
Feminino	12-14	3.314	343.871	2,2	1,3-4,0
	15-17	3.722	325.047	2,3	1,7-3,3
Masculino	12-14	2.160	350.923	2,7	1,8-4,1
	15-17	2.465	317.835	3,4	2,6-4,4
Centro-Oeste		5.441	525.340	2,2	1,7-30
Feminino	12-14	1.543	123.669	1,9	1,1-3,1
	15-17	1.802	140.324	2,1	1,4-3,3
Masculino	12-14	1.060	124.867	3,2	1,8-5,4
	15-17	1.056	136.480	1,8	1,1-3,0
Sudeste		8.460	3.488.424	2,4	1,9-3,0
Feminino	12-14	2.141	755.994	1,9	1,0-3,7
	15-17	3.024	995.770	1,9	1,1-3,1
Masculino	12-14	1.459	760.343	2,7	1,8-4,0
	15-17	1.836	976.317	3,1	2,0-4,8
Sul		4.690	821.105	3,5	2,6-4,9
Feminino	12-14	1.157	186.354	5,7	2,5-12,7
	15-17	1.538	226.094	2,1	1,0-4,3
Masculino	12-14	879	188.266	1,6	0,9-2,8
	15-17	1.116	220.391	4,8	3,3-6,9

A capital brasileira que apresentou a maior prevalência de SM foi Belém (3,8% [IC95% 2,7-5,2]), e a menor, Macapá (0,9% [IC95% 0,3-2,7]), ambas na região Norte, macrorregião que apresentou as maiores variações entre suas capitais (Figura 2). Entretanto, considerando todos os estratos da amostra, a maior prevalência de SM ocorreu nos municípios do interior da macrorregião Sul (4,1% [IC95% 2,8-5,9]).

**Figura 2:** Prevalências (%) e IC95% de síndrome metabólica nos adolescentes, segundo estratos de capitais e do interior das regiões. ERICA, Brasil, 2013-2014.

A prevalência de SM em escolas públicas foi 2,8% (IC95% 2,4-3,2) e nas escolas privadas, 1,9% (IC95% 1,4-2,4). Nas escolas públicas, a prevalência nos adolescentes do sexo masculino foi 3,1% (IC95% 2,5-3,7) e nos do sexo feminino, 2,6% (IC95% 2,0-3,3). Nas escolas privadas, foi menor tanto em adolescentes do sexo masculino (2,5% [IC95% 1,9-3,3]), quanto do sexo feminino (1,3% [IC95% 0,9-1,9]).

As prevalências de SM nos adolescentes com estado nutricional adequado foram inferiores a 0,2%, tanto no sexo masculino (0,008% [IC95% 0,003-0,03]) como no feminino (0,1% [IC95% 0,05-0,4]). Nas regiões Norte, Nordeste e Sudeste a prevalência de SM neste grupo de adolescentes foi de 0,1%, e ainda menor nas regiões Centro-Oeste e Sul.

A Tabela 3 mostra as prevalências da SM de adolescentes com sobrepeso ou obesidade, por região e sexo. Em adolescentes com obesidade, a prevalência de síndrome metabólica foi mais de seis vezes maior do que naqueles com sobrepeso. Essa proporção foi sempre maior nos adolescentes do sexo masculino, com exceção da região Sul, na qual também se observou a maior razão entre as regiões. As prevalências de SM e IC95% por estado nutricional, segundo sexo ou faixa etária no Brasil e nas macrorregiões podem ser consultadas na Tabela 4.

**Tabela 3:** Prevalências (%) e IC95% de síndrome metabólica em adolescentes com sobrepeso ou obesidade, segundo sexo e macrorregião. ERICA, Brasil, 2013-2014.

Regiões/Sexo	Sobrepeso		Obesidade	
	
	%	IC95%	%	IC95%
Brasil	3,3	2,4-4,5	21,3	18,5-24,5
Feminino	4,0	2,6-6,0	17,5	13,7-21,9
Masculino	2,6	1,6-4,1	24,5	20,1-29,6
Norte	3,3	2,4-4,4	24,7	20,5-29,5
Feminino	5,1	3,6-7,1	22,4	16,6-29,5
Masculino	1,3	0,7-2,6	26,3	20,1-33,6
Nordeste	3,3	2,1-5,3	21,7	16,7-27,8
Feminino	3,5	2,2-5,6	20,1	12,2-31,2
Masculino	3,1	1,7-5,7	22,8	17,9-28,6
Centro-Oeste	2,1	1,2-3,0	21,7	16,1-27,9
Feminino	3,1	1,9-5,1	23,9	16,2-33,8
Masculino	1,2	0,5-2,4	20,5	14,8-27,7
Sudeste	3,9	2,4-6,2	18,9	14,5-24,2
Feminino	4,8	2,5-9,1	11,3	7,8-16,0
Masculino	3,0	1,4-6,1	26,1	17,9-36,4
Sul	1,6	0,9-3,0	27,3	20,7-35,2
Feminino	1,8	0,8-4,0	31,8	19,4-47,5
Masculino	1,4	0,6-3,4	23,6	16,5-32,6

**Tabela 4:** Prevalências (%) e IC95% de síndrome metabólica em adolescentes, por sexo, idade e regiões brasileiras de acordo com o estado nutricional. ERICA, 2013-2014.

Característica	Estado nutricional	%	IC95%
Brasil	Eutrófico	0,1	0,02-0,2
	Sobrepeso	3,3	2,4-4,5
	Obeso	21,3	18,5-24,5
Feminino	Eutrófico	0,1	0,03-0,4
	Sobrepeso	4,0	2,6-6,0
	Obeso	17,5	13,7-21,9
Masculino	Eutrófico	0,008	0,003-0,03
	Sobrepeso	2,6	1,6-4,1
	Obeso	24,5	20,1-29,6
12-14 anos	Eutrófico	0,01	0,006-0,04
	Sobrepeso	1,7	0,7-3,8
	Obeso	19,6	15,8-24,1
15-17 anos	Eutrófico	0,1	0,04-0,4
	Sobrepeso	4,9	3,6-6,6
	Obeso	23,5	19,1-28,6
Norte	Eutrofico	0,1	0,02-0,2
	Sobrepeso	3,3	2,4-4,4
	Obeso	24,7	20,5-29,5
Nordeste	Eutrófico	0,1	0,03-0,2
	Sobrepeso	3,3	2,1-5,3
	Obeso	21,7	16,89-27,5
Centro-Oeste	Eutrófico	0,02	0,01-0,1
	Sobrepeso	1,6	0,9-3,0
	Obeso	27,3	20,7-35,2
Sudeste	Eutrófico	0,02	0,0-0,1
	Sobrepeso	2,1	1,4-3,2
	Obeso	21,7	16,7-27,8
Sul	Eutrófico	0,1	0,01-0,4
	Sobrepeso	3,9	2,5-6,2
	Obeso	18,9	14,5-24,2

Na Figura 3 observa-se que, em todos os estratos da amostra, a prevalência de SM foi maior nos adolescentes obesos do que nos não obesos. As prevalências de SM e intervalos de confiança nos estratos da amostra podem ser consultadas na Tabela 5.

**Figura 3:** Prevalências (%) e IC95% de síndrome metabólica em adolescentes sem obesidade e com obesidade segundo estratos de capitais e do interior das regiões. ERICA, Brasil, 2013-2014.

**Tabela 5:** Prevalências (%) e IC95% de síndrome metabólica em adolescentes por capitais e estratos do interior das regiões. ERICA, Brasil, 2013-2014.

Estratos	Total		Sem obesidade		Com obesidade	
		
	%	IC95%	%	IC95%	%	IC95%
Interior Norte	1,8	1,3-2,5	0,7	0,4-1,1	18,4	12,2-26,9
Porto Velho	3,3	1,8-5,7	0,6	0,3-1,6	32,6	19,6-49,0
Rio Branco	1,9	1,0-3,3	0,3	0,1-1,0	19,6	12,9-28,6
Manaus	2,2	1,5-3,2	0,5	0,3-1,1	21,1	13,2-31,8
Boa Vista	1,7	0,6-5,0	0,2	0-0,8	20,1	7,2-45,1
Belém	3,8	2,7-5,2	1,0	0,6-1,5	37,3	27,2-48,7
Macapá	0,9	0,3-2,7	0,1	0-0,5	22,6	6,6-54,6
Palmas	2,1	1,0-4,3	0,3	0,1-1,1	28,4	16,1-45,2
Interior Nordeste	2,9	2,2-3,8	0,9	0,5-1,8	21,3	13,0-32,9
São Luís	2,4	1,5-3,7	0,4	0,2-0,8	36,5	23,1-52,5
Teresina	1,6	0,8-3,2	0,2	0,1-0,8	20,6	10,6-36,3
Fortaleza	2,6	1,7-3,8	0,4	0,2-0,9	23,4	15,8-33,3
Natal	2,3	1,2-4,1	1,0	0,4-2,6	14,4	8,3-23,6
João pessoa	3,0	1,8-4,9	0,5	0,1-1,8	36,2	19,5-57,1
Recife	2,7	1,9-3,9	0,8	0,3-1,8	19,3	13,7-26,6
Maceió	2,2	1,3-3,7	0,4	0,2-1,0	23,8	12,8-40,0
Aracaju	2,2	1,4-3,6	0,3	0,1-1,1	19,5	11,2-31,7
Salvador	2,6	1,5-4,3	0,8	0,4-1,4	19,9	10,1-35,5
Interior Centro-Oeste	2,8	1,6-4,8	0,4	0,2-0,9	30,6	19,7-44,3
Campo Grande	2,2	1,2-4,2	0,4	0,1-1,3	18,3	9,4-32,6
Cuiabá	2,6	1,7-3,9	1,1	0,7-1,7	21,2	10,9-37,3
Goiânia	2,1	1,2-3,8	0,3	0,1-1,0	18,4	9,7-32,2
Brasília	1,7	1,1-2,6	0,3	0,1-0,7	17,0	11,2-24,8
Interior Sudeste	2,6	2,0-3,5	1,0	0,5-1,8	20,6	14,4-28,6
Belo Horizonte	1,3	0,8-1,9	0,6	0,3-1,1	10,8	6,2-18,2
Vitória	2,5	1,5-4,2	0,8	0,3-2,0	21,3	10,8-37,5
Rio de Janeiro	1,9	1,3-2,7	0,5	0,2-1,1	11,6	7,5-17,7
São Paulo	2,1	1,5-3,0	0,5	0,3-1,1	20,2	12,9-30,4
Interior Sul	4,1	2,8-5,9	0,2	0,1-0,7	30,0	22,1-39,3
Curitiba	2,0	1,3-3,2	0,9	0,3-2,2	16,3	9,7-26,0
Florianópolis	1,8	0,8-4,0	0,1	0,0-0,7	25,0	9,7-50,7
Porto Alegre	2,2	1,3-3,6	0,8	0,3-2,5	13,9	8,7-21,5

Em relação à composição da SM, que de acordo com o critério adotado incluiu obrigatoriamente a presença da CC elevada, foi observado que 82,3% (IC95% 79,2-85,0) dos adolescentes com SM apresentaram três componentes presentes, 15,6% (IC95% 13,0-18,6) tinham quatro componentes, e 2,1% (IC95% 1,0-4,5) tinham cinco componentes.

As combinações de componentes da SM mais frequentemente observadas foram: CC elevada, HDL-c baixo e pressão arterial elevada em 33,4%; CC elevada, HDL-c baixo e triglicerídeos elevados em 31,8%; e CC elevado, HDL-c baixo e pressão arterial e triglicerídeos elevados em 9,5%. Estas combinações de fatores foram responsáveis por cerca de 3/4 de todas as combinações possíveis.

A Tabela 6 descreve as prevalências dos componentes da SM em toda a amostra, a prevalência de SM naqueles com cada um dos componentes e a prevalência de cada componente nos adolescentes com SM. Cerca de 1/3 dos adolescentes tiveram HDL-c baixo, o que se refletiu na prevalência elevada desse componente dentre os adolescentes com SM; porém, menos de 10,0% dos adolescentes com essa alteração tinham SM. Quanto aos triglicerídeos elevados, apesar de apresentarem prevalência baixa comparada aos demais componentes na amostra total, estavam presentes em quase metade dos adolescentes com SM; e a prevalência de SM naqueles com esta alteração foi alta (26,8%), maior até do que naqueles com o componente obrigatório, a CC elevada (20,5%). A glicose elevada, que teve a prevalência mais baixa dos componentes na amostra total, teve sua presença associada com prevalência cerca de duas vezes maior de SM do que na presença do HDL-c baixo. Quase 20,0% dos adolescentes com pressão arterial elevada, pelos critérios da IDF, foram classificados com SM; e dentre os adolescentes com SM, pouco mais da metade apresentaram pressão arterial elevada (Tabela 6).

**Tabela 6:** Prevalências (%) e IC95% dos componentes da SM na população, de SM nos adolescentes com componentes particulares, e dos componentes nos adolescentes com SM. ERICA, Brasil, 2013-2014.

Componentes da SM	Prevalência do componente		Prevalência de SM nos adolescentes com o componente		Prevalência do componente nos adolescentes com SM
		
	%	IC95%	%	IC95%	%
CC^a^	12,6	11,6-13,7	20,5	18,2-22,9	100
HDL-c^b^	32,7	30,3-35,2	7,2	6,3-8,2	90,7
Triglicerídeos^a^	4,6	4,1-5,1	26,8	22,4-31,7	47,6
Glicose^a^	4,1	3,5-4,8	15,0	10,8-20,4	20,6
Pressão arterial^a^	8,2	7,6-8,9	18,7	15,6-22,1	57,6

SM: síndrome metabólica; CC: circunferência da cintura; HDL-c: HDL-colesterol

^a^ Elevado.

^b^ Baixo.

## DISCUSSÃO

Este estudo foi o primeiro realizado em amostra representativa da população brasileira nessa faixa etária, apresentando informações importantes quanto à prevalência de SM. As prevalências observadas variaram de acordo com a macrorregião, sexo e idade, sem apresentar um padrão específico único. A prevalência de SM na região Sul foi maior do que nas demais regiões, em boa parte devido à prevalência observada nos municípios de mais de 100 mil habitantes do interior dessa macrorregião. Esse dado pode indicar diferentes hábitos alimentares e de estilo de vida em relação às macrorregiões, uma vez que esses são os principais fatores na gênese da obesidade, componente central no diagnóstico da SM.

A observação de prevalência de SM maior nos adolescentes das escolas públicas indica uma possível associação de fatores socioeconômicos com a SM. A análise de características que possam variar de acordo com o nível socioeconômico, tais como educação dos pais, hábitos alimentares ou prática de atividades físicas podem contribuir para a compreensão dessas relações. Alguns estudos observaram uma relação oposta ao encontrado no ERICA, ou seja, prevalência de SM maior naqueles com maior renda^5^ ^,^ ^7^ ^,^ ^8^ ^.^

Existem mais de 40 definições de SM para crianças e adolescentes^11^ ^.^ A definição de SM proposta pelo IDF exibe prevalências que costumam ser mais baixas do que as estimadas com as outras definições de uso frequente. Alguns estudos com proporções menores do que o ERICA, como o de Alvarezet et al.^1^, que avaliaram 577 adolescentes escolares da rede pública e privada no município de Niterói, RJ, observaram prevalência de 1,6% (IC95% 0,6-3,9) com a definição da IDF e de 6,0% (IC95% 3,0-7,8) com os critérios do NCEP-ATPIII. A presença obrigatória da CC elevada na definição da IDF pode ter sido responsável por essa diferença.

Não há consenso sobre os critérios mais adequados, e os adolescentes são uma fração populacional, cuja principal característica é estar em transformação. Portanto, o uso de uma definição mais específica (que não rotule como portadores de SM aqueles com baixa probabilidade de terem esta síndrome ou seus falsos-positivos) parece mais apropriado do que o uso de um critério mais sensível.

No ERICA, a prevalência de SM entre os obesos diferiu quanto aos sexos somente nas regiões Norte e Sudeste. Na primeira, foi maior entre os adolescentes do sexo feminino com sobrepeso; e na segunda, no sexo masculino, nos obesos. A maioria das pesquisas relata maior prevalência nos adolescentes do sexo masculino^21^ ^,^ ^23^.

Utilizando dados sobre adolescentes do *National Health and Nutrition Examination Survey* (NHANES), Laurson et al.^13^ observaram que a prevalência de SM, segundo os critérios do NCEP-ATPIII, em 1.785 adolescentes do sexo masculino com estado nutricional adequado foi 0,8% (IC95% 0,1-1,5), naqueles com sobrepeso foi 6,8% (IC95% 1,2-12,4) e, em obesos, 35,4% (IC95% 27,1-43,7). Em 1.600 adolescentes do sexo feminino, a prevalência observada naquelas sem sobrepeso ou obesidade foi 1,7% (IC95% 0,1-3,3), naquelas com sobrepeso foi 9,2% (IC95% 4,4-14,0) e nas obesas, 24,6% (IC95% 17,2-31,9). No presente estudo, a prevalência de SM foi menor nos adolescentes de ambos os sexos, independente do estado nutricional, quando comparada às prevalências no estudo de Laurson et al., porém os critérios utilizados foram diferentes. Nasreddine et al^16^, usando os critérios da IDF, observaram prevalência de 21,2% em adolescentes libaneses obesos, 3,8% naqueles com sobrepeso e 1,2% naqueles sem sobrepeso ou obesidade, resultados semelhantes aos do ERICA.

Neste estudo, as prevalências dos componentes da SM observadas nos adolescentes com SM, em ordem decrescente, foram: CC elevada, HDL-c baixo, pressão arterial, triglicerídeos e glicose elevados. Nasreddine et al.^16^ observaram ordem semelhante. No entanto, a análise da participação de cada componente da SM permite visualizar a importância de modo individualizado. Embora a prevalência do HDL-c baixo isoladamente tenha sido a mais elevada, nos adolescentes com triglicerídeos ou pressão arterial elevados a prevalência de SM foi maior. Ou seja, segundo os critérios da IDF, o triglicerídeos foi um componente relevante.

A SM está diretamente associada à resistência insulínica e esta é capaz de promover e explicar o surgimento, a prevalência e a magnitude de cada um dos componentes da SM^3^ ^,^ ^14^ ^,^ ^19^. A presença de apenas um dos componentes da SM justifica intervenções não medicamentosas, tais como promoção de hábitos de vida saudáveis, além de acompanhamento em serviços de saúde. Embora as prevalências observadas no ERICA sejam baixas, a forte associação observada com o estado nutricional indica que a prevenção da obesidade pode ter um impacto significativo na diminuição da prevalência da SM e de suas complicações cardiovasculares na vida adulta.

## Footnotes

a Instituto Brasileiro de Geografia e Estatística. Projeção da população do Brasil por sexo e idade – 1980-2050: revisão 2008. Rio de Janeiro (RJ): IBGE; 2008 [citado 2015 set]. (Estudos e pesquisas, 24). Disponível em: http://www.ibge.gov.br/home/estatistica/populacao/projecao_da_populacao/2008/projecao.pdf

b Stata Corp. Stata statistical software: release 14. College Station, Texas; 2015.
